# Supplementary material for: Automated hippocampal unfolding for morphometry and subfield segmentation with HippUnfold
Source: eLife. 2022 Dec 15;11:e77945. doi: 10.7554/eLife.77945 (PMC9831605; doi:10.7554/eLife.77945)
Supplement: Supplementary file 4. [file elife-77945-supp4.pdf]

HippUnfold Algorithmic Details

HippUnfold Development Team

Nov 26, 2022

TABLE OF CONTENTS

1 Hippocampal unfolding **1**

- 1.1 Template-based shape injection . . . 1
- 1.2 Fast marching initialization . . . 2
- 1.3 Solving Laplace's equation . . . 2
- 1.4 Equivolumetric laminar coordinates . . . 3
- 1.5 Warps for unfolding . . . 3
  - 1.5.1 Forward warps . . . 3
    - 1.5.1.1 Warps for surfaces and images . . . 3
- 1.6 Inverse warps . . . 4
- 1.7 Standard surface meshes . . . 4
- 1.8 Subfield segmentation . . . 5
- 1.9 Dentate gyrus unfolding . . . 5

CHAPTER

ONE

HIPPOCAMPAL UNFOLDING

Our approach to unfolding the hippocampus involves constructing a coordinate system, defined using the solutions to partial differential equations to enforce smoothness, and to employ anatomically-derived boundary conditions. Each of the three coordinates (AP, PD, IO) are solved independently of each other, each using distinct boundary conditions defined by the hippocampus tissue segmentation. With the notation L_{ROI} to represent the labelled set of voxels in the hippocampus of a specific ROI, the domain of the solution, along with boundary conditions as source and sink, are defined as follows:

$$L_{domain} = \begin{cases} L_{GM} \cup L_{DG}, & \text{if coords} = AP \vee PD \vee IO \end{cases}$$

$$L_{source} = \begin{cases} L_{HATA}, & \text{if coords} = AP \\ L_{MTLC}, & \text{if coords} = PD \\ L_{SRLM} \cup L_{Pial} \cup L_{Cyst}, & \text{if coords} = IO \end{cases}$$

$$L_{sink} = \begin{cases} L_{IndGris}, & \text{if coords} = AP \\ L_{DG}, & \text{if coords} = PD \\ L_{background}, & \text{if coords} = IO \end{cases}$$

1.1 Template-based shape injection

We make use of a fluid diffeomorphic image registration, between a template hippocampus tissue segmentation, and the U-net tissue segmentation, in order to 1) help enforce the template topology, and 2) provide an initialization to the Laplace solution. By performing a fluid registration, driven by the segmentations instead of the MRI images, the warp is able to bring the template shape into close correspondence with the subject, but the regularization helps ensure that the topology present in the template is not broken. The template we use was built from 22 *ex vivo* images from the Penn Hippocampus Atlas.

The registration is performed using greedy, initialized using moment tensor matching (without reflections) to obtain an affine transformation, and a multi-channel sum of squared differences cost function for the fluid registration. The channels are made up of binary images, split from the multi-label tissue segmentations, which are then smoothed with a Gaussian kernel with standard deviation of $0.5mm$. The Cyst label is replaced by the SRLM prior to this, since the locations of cysts are not readily mapped using a template shape. After warping the discrete template tissue labels to the subject, the subject's Cyst label is then re-combined with the transformed template labels.

The pre-computed Laplace solutions on the template image (analogous to method described below), $\psi^{template}_{A\to P}$, $\psi^{template}_{P\to D}$, $\psi^{template}_{I\to O}$, are then warped to the subject using the diffeomorphic registration to provide an initialization for the subject.

1.2 Fast marching initialization

As an alternative if template-based shape injection is not used, we employ a fast marching method to provide an initialization to the Laplace solution, to speed up convergence. We make use of the scikit-fmm Python package, that finds approximate solutions to the boundary value problems of the Eikonal equation,

$$F(\mathbf{x})\,|\nabla\phi(\mathbf{x})| = 1,$$

which describes the evolution of a closed curve as a function of time, ϕ, with speed $F(\mathbf{x}) > 0$ in the normal direction at a point $\mathbf{x}$ on the curve. The fast marching implementation provides a function (image) representing travel time to the zero contour of an input, ϕ.

We first perform fast marching from the source (forward direction), by initializing the zero contour with:

$$\phi_0(\mathbf{x}) = \begin{cases} 0, & \mathbf{x} \in L_{source} \\ 1, & \mathbf{x} \notin L_{source} \end{cases}$$

and we make use of the NumPy masked arrays to avoid computations in voxels outside of L_{domain}. We use a constant speed function of 1, and perform fast marching to produce a travel-time image, $T_{forward}(\mathbf{x})$, that is normalized by $\max(T_{forward}(\mathbf{x}))$ to obtain an image from 0 to 1 (0 at the *source*). We perform the same process for the *sink* region, by setting ϕ based on L_{sink}, which produces a normalized $T_{backward}(\mathbf{x})$ image. We combine forward and backward images by averaging $T_{forward}$ and $1 - T_{backward}$ to produce the combined fast marching image, $T_{fastmarch}$.

1.3 Solving Laplace's equation

Laplace's equation is a second-order partial differential equation,

$$\nabla^2\psi(\mathbf{x}) = 0,$$

where ψ is a scalar field enclosed between the source and sink boundaries. A simple approach to solve Laplace's equation is with an iterative finite-differences approach (Jacobian method), where each voxel in the field is updated at each iteration as the average of the neighbouring grid points, e.g. for a 2-D field,

$$\psi_{i+1}(x,y) = \frac{1}{4}\left[\psi_i(x+\Delta x, y) + \psi_i(x-\Delta x, y) + \psi_i(x, y+\Delta y) + \psi_i(x, y-\Delta y)\right].$$

For our 3-D implementation, we use the nearest 18 neighbours, and perform the operation using convolution with a kernel size of $3 \times 3 \times 3$, or 27 voxels. We initialize the ψ field as follows:

$$\psi_{i=0}(\mathbf{x}) = \begin{cases} 0, & \mathbf{x} \in L_{source} \\ 1, & \mathbf{x} \in L_{sink} \\ T_{fastmarch}(\mathbf{x}), & \mathbf{x} \in L_{domain} \\ NaN, & \text{otherwise.} \end{cases}$$

We used the convolve method from the AstroPy Python package instead of NumPy's convolve, because it avoids using NaN values (i.e. voxels outside the gray matter) in the convolution, replacing them with interpolated values using the convolution kernel. We iteratively update ψ until either the sum-of-squared-differences, $\sum\left[\psi_i(\mathbf{x}) - \psi_{i-1}(\mathbf{x})\right]^2$, is less than 1×10^{-5}, or a maximum iterations of 10,000 are reached. Note that more efficient approaches to solving Laplace's equation are possible (such as successive over-relaxation), however, we used this more conservative approach to avoid stability and convergence issues.

We use this approach to independently produce $\psi_{A\to P}$ and $\psi_{P\to D}$. Note that because we are solving these fields independent of one another, their gradient fields are not guaranteed to be perpendicular, however, we have not observed large deviations in practice. A solution for jointly solving $\psi_{A\to P}$ and $\psi_{P\to D}$ is left for future work.

1.4 Equivolumetric laminar coordinates

For the laminar, or inner-outer coordinates, , $\psi_{I\to O}$, it has been shown that an equivolumetric approach, that preserves the volume of cortical segments by altering laminar thickness based on the curvature, is more anatomically-realistic for the cerebral cortex. We implement this approach as the default for the IO coordinates, making use of the implementation in NighRes. Here, we set the inner level-set to be L_{source}, effectively the SRLM, and the outer level-set as the entire hippocampus. The continuous depth image returned by the volumetric layering function is then used directly as $\psi_{I\to O}$.

1.5 Warps for unfolding

We make use of the three coordinates, $\psi_{A\to P}$, $\psi_{P\to D}$, and $\psi_{I\to O}$, to create 3D warp fields that transform images and surfaces between the native domain $D_{native} \subset \mathbf{R}^3$, and the unfolded domain $D_{unfolded} \subset \mathbf{R}^3$.

Because solve Laplace's equation in voxels restricted to the gray matter, the native domain, D_{native} is made up of $\mathbf{x} = (x, y, z)$, where $\mathbf{x} \in L_{GM}$.

The unfolded domain, $D_{unfolded}$, is a distinct 3D space, indexed by $\mathbf{u} = (u, v, w)$, where $u = \psi_{A\to P}(x, y, z)$, $v = \psi_{P\to D}(x, y, z)$, and $w = \psi_{I\to O}(x, y, z)$. The ψ fields are initially normalized to $0 \to 1$, which would produce a rectangular prism between $(0, 0, 0)$ and $(1, 1, 1)$. However, we have re-scaled the aspect ratio and discretization to better approximate the true size of the hippocampus along each dimension, producing a volume of size $256x128x16$. To facilitate visualization, we set the origin to $(0, 200, 0)$ (in *mm*) so as not to overlap with our native space) and set a physical voxel spacing of 0.15625*mm* in each direction.

1.5.1 Forward warps

The transformation, or displacement warp field, that takes points, $\mathbf{x} \in \mathbf{R}^3$, (or surfaces) from native to unfolded space, is denoted as $T^{surf}_{\mathbf{x}\to\mathbf{u}} : (x, y, z) \to (u, v, w)$, and is simply defined as:

$$T^{surf}_{\mathbf{x}\to\mathbf{u}}(x, y, z) = (\psi_{A\to P}(x, y, z) - x, \psi_{P\to D}(x, y, z) - y, \psi_{I\to O}(x, y, z) - z)\,,$$

and is valid for any point, or surface vertex, within the native domain, D_{native}. Note that construction of this displacement field also involves rescaling for the physical voxel dimensions of the unfolded domain as described above, which is left out of the above equations.

1.5.1.1 Warps for surfaces and images

The warp field that transforms points/surfaces from native to unfolded also transforms images from the unfolded to the native domain,

$$T^{surf}_{\mathbf{x}\to\mathbf{u}} = T^{img}_{\mathbf{u}\to\mathbf{x}},$$

since images on a rectilinear grid must be warped with the inverse of the transformation that is required for points or surfaces. This is not particular to HippUnfold, and is true for any transformations. This is because instead of pushing forward from the moving image grid (which leads to off-grid locations), we start at the fixed grid-point (e.g. in native space), and pull-back with the inverse transformation to determine an (off-grid location) in unfolded space, to interpolate image intensities from neighbouring grid locations (e.g. in the unfolded space).

1.6 Inverse warps

To obtain, $T^{surf}_{\mathbf{u}\to\mathbf{x}} : (u, v, w) \to (x, y, z)$, or equivalently, $T^{img}_{\mathbf{u}\to\mathbf{x}} : D_{native} \to D_{unfolded}$, requires determining the inverse of the transformation that is provided by the ψ fields. We achieve this by first applying the forward transformation on all grid locations in the native domain, obtaining

$$T^{surf}_{\mathbf{x}\to\mathbf{u}}(x, y, z) = (Tx, Ty, Tz)\,, \qquad \forall (x, y, z) \in D_{native}.$$

The source native grid location, (x, y, z) for each the transformed points (Tx, Ty, Tz) is used to define the inverse transformation:

$$T^{scattered}_{\mathbf{u}\to\mathbf{x}}(Tx, Ty, Tz) = (x - Tx, y - Ty, z - Tz)$$

However, these points are only defined at scattered locations in the unfolded space, thus we need to use interpolation between these points to obtain $T^{surf}_{\mathbf{u}\to\mathbf{x}}$ defined at all grid locations in $D_{unfolded}$. We perform this operation using the *griddata* function from *SciPy*, which interpolates unstructured multi-variate data onto a grid, by triangulating the input data with Qhull, then performing piecewise linear barycentric interpolation on each triangle. Due to discretization in the ψ fields that produced the forward transformation, there are voxels outside the convex hull of the points that are not able to be linearly interpolated. To fill these values in, we make use of the *griddata* function with nearest neighbour interpolation instead. Note that this produces singularities in the warp (since points outside the convex hull have the same destination as the nearest convex hull point, but this is strictly limited to the edges of the hippocampus, and have little practical implications in our experience. After linear and nearest neighbour interpolation, the final warp field is produced:

$$T^{surf}_{\mathbf{u}\to\mathbf{x}} = T^{img}_{\mathbf{x}\to\mathbf{u}}.$$

Altogether, this provides transformations to warp either images or surfaces, in either direction (that is, native to unfolded, or unfolded to native). Image warps are defined using ITK format standards (Left-posterior-superior, or LPS coordinate system), and thus are compatible with existing tools (e.g. ANTS) to perform the transformation, or to concatenate transforms. The surface warps use a different coordinate system (Right-anterior-superior, or RAS coordinate system), for compatibility with the Connectome Workbench *surface-apply-warpfield* function, that operates on GIFTI files.

1.7 Standard surface meshes

Since the unfolding produces individual warps that can be used to transform surfaces from the unfolded domain to any individual native domain, we can produce a standardized mesh in the unfolded space (e.g. spanning a 2-D plane at a constant $w = C$ laminar level), and transform this to each hippocampus to generate a native-space hippocampal surface mesh, with 1-1 correspondence in vertices across hippocampi.

Our previous work made use of a spatially-uniform triangulated mesh in the unfolded space, now referred to as the *unfoldiso* mesh. Triangles in this mesh have equal size in the unfolded domain, however, when transformed to a subject's native space, distortions in triangle size are produced. To address this, we triangulated surfaces with an locally-adaptive number of points, where the spacing of the points was calculated to obtain approximately equal vertex spacing once transformed to the native space. The surface areas and vertex spacing were optimized on the Human Connectome Project Unrelated 100 subset, by transforming the *unfoldiso* surface, calculating the average area and spacing over all 100 subjects, then generating a range of triangular meshes with adaptive spacing. We selected meshes with mean vertex spacing close to 2mm, 1mm, and 0.5mm for our standard meshes.

1.8 Subfield segmentation

Subfield atlases in HippUnfold are now defined in the volumetric unfolded space, and are propagated to individual images or surfaces with distinct methods. For surface meshes, the subfield volumetric labels are sampled on a standard surface mesh using the Connectome Workbench function *volume-to-surface-mapping*, which creates a label GIFTI file for the surface vertices that is applicable to both unfolded and native surfaces. For volumetric images, it is possible to simply apply $T^{img}_{\mathbf{u}\rightarrow\mathbf{x}}$ to the subfield atlas. The current workflow applies an analogous approach, using $\psi_{A\rightarrow P}$ and $\psi_{P\rightarrow D}$ to interpolate, as our subfield atlas labels historically existed as surface labels, instead of the current volumetric labels, but both approaches should yield the same result.

The volumetric subfield labels are then modified to override the L_{SRLM}, L_{Cyst}, and L_{DG} labels from the tissue segmentation, since these labels are not included in the subfield atlas.

1.9 Dentate gyrus unfolding

Unfolding for the dentate gyrus conceptually identical to the hippocampus, however, the $\psi_{I\rightarrow O}$ and $\psi_{P\rightarrow D}$ fields are swapped, since the dentate gyrus tissue is topologically-perpendicular to the rest of the hippocampus.

Furthermore, because the dentate gyrus is a much smaller structure than the hippocampus, solving Laplace's equation for each individual hippocampus can be challenging if the spatial resolution is limited. Thus instead, we solely make use of the template shape injection, and use the pre-computed Laplace solution, $\psi^{template}$, to define the coordinates. Also, for the pre-computed solution, the $\psi_{A\rightarrow P}$ field is computed from the hippocampus (since this coordinate is naturally constrained to be identical for both structures).
